# Non-Smoking Male Adolescents' Reactions to Cigarette Warnings

**DOI:** 10.1371/journal.pone.0065533

**Published:** 2013-08-07

**Authors:** Jessica K. Pepper, Linda D. Cameron, Paul L. Reiter, Annie-Laurie McRee, Noel T. Brewer

**Affiliations:** 1 Gillings School of Global Public Health, University of North Carolina, Chapel Hill, North Carolina, United States of America; 2 Lineberger Comprehensive Cancer Center, University of North Carolina, Chapel Hill, North Carolina, United States of America; 3 Psychological Sciences, School of Social Sciences, Humanities, and Arts, University of California, Merced, California, United States of America; 4 Division of Cancer Prevention and Control, College of Medicine, The Ohio State University, Columbus, Ohio, United States of America; 5 Department of Pediatrics, University of Minnesota, Minneapolis, Minnesota, United States of America; Fundación para la Prevención y el Control de las Enfermedades Crónicas No Transmisibles en América Latina (FunPRECAL), Argentina

## Abstract

**Background:**

The U.S. Food and Drug Administration (FDA) is working to introduce new graphic warning labels for cigarette packages, the first change in cigarette warnings in more than 25 years. We sought to examine whether warnings discouraged participants from wanting to smoke and altered perceived likelihood of harms among adolescent males and whether these warning effects varied by age.

**Methods:**

A national sample of 386 non-smoking American males ages 11–17 participated in an online experiment during fall 2010. We randomly assigned participants to view warnings using a 2×2 between-subjects design. The warnings described a harm of smoking (addiction or lung cancer) using text only or text plus an image used on European cigarette package warnings. Analyses tested whether age moderated the warnings' impact on risk perceptions and smoking motivations.

**Results:**

The warnings discouraged most adolescents from wanting to smoke, but lung cancer warnings discouraged them more than addiction warnings did (60% vs. 34% were “very much” discouraged, *p*<.001). Including an image had no effect on discouragement. The warnings affected several beliefs about the harms from smoking, and age moderated these effects. Adolescents said addiction was easier to imagine and more likely to happen to them than lung cancer. They also believed that their true likelihood of experiencing any harm was lower than what an expert would say.

**Conclusions:**

Our findings suggest that warnings focusing on lung cancer, rather than addiction, are more likely to discourage wanting to smoke among adolescent males and enhance their ability to imagine the harmful consequences of smoking. Including images on warnings had little effect on non-smoking male adolescents' discouragement or beliefs, though additional research on the effects of pictorial warnings for this at-risk population is needed as the FDA moves forward with developing new graphic labels.

## Introduction

To address harms caused by tobacco use, which is the leading cause of preventable deaths worldwide [1], the World Health Organization calls for the implementation of large warning labels on tobacco products [2]. Many nations have already adopted this approach [3]. In November 2010, the U.S. Food and Drug Administration (FDA) proposed new graphic warning labels for cigarette packages [4]. Although the implementation of these specific graphic warnings was blocked by the court, the FDA will propose new graphic labels in the future as required by the Family Smoking Prevention and Tobacco Control Act [5]–[7].

The FDA's previously proposed warnings included both images and text, and future warnings will do the same [6]. Surveys, as well as some experimental studies with non-U.S. populations or current smokers, indicate that warnings with images are generally more effective for increasing motivation to not smoke or attempt quitting than text-only warnings [8]. However, at least one experimental study suggests that this finding may not hold true for non-smoking U.S. adolescents [9].

The previous FDA labels, and most likely future labels as well, contain warnings about different harms from smoking, including lung cancer and addiction, both of which are addressed by warning labels currently in use in other countries. Adolescents may perceive some harms as more salient than others. Lung cancer, a concrete medical consequence that may elicit specific mental images (e.g., blackened lungs and oxygen tanks), might be easier for adolescents to appreciate than addiction, a more abstract concept that may not call to mind specific images. On the other hand, adolescents tend to discount long-term consequences and place more weight on short-term outcomes compared to adults, and thus they might be less concerned about lung cancer, a long-term consequence, than addiction, a short-term consequence [10]. These two harms also vary in their applicability to adolescents. Addiction develops quickly after smoking initiation and could affect teen smokers personally [11]. Lung cancer takes longer to develop, so it is unlikely to affect teen smokers in the immediate aftermath of smoking initiation.

Ability to understand harms could also vary by the age of the adolescent [12], [13]. For example, between ages 10 and 18, children become more likely to anticipate the consequences of their actions [10]. Beliefs about the addictive properties of smoking decrease between ages 11–14, but begin to increase between ages 15–18, and beliefs that smoking could be personally harmful follow the same pattern during these years [14]. Thus, warnings that focus on different types of consequences (e.g., short-term versus long-term, addiction versus other harms) of smoking may vary in their impact on smoking risk beliefs and motivations for adolescents of different ages.

Because most smokers begin smoking during adolescence and males are more likely to use tobacco products than females [15], it is important to understand how male adolescents respond to cigarette package warnings. The purpose of this study was to examine whether different types of warnings discouraged wanting to smoke and altered perceived likelihood of harms among adolescent males. To distinguish adolescents' risk judgments about themselves from other risk judgments, we asked about their own beliefs about the likelihood of experiencing harms as well as what they thought an expert would say about the likelihood. We also sought to understand the role of age in adolescents' reactions to the warnings. We predicted that warnings would increase the perceived likelihood of smoking-related harms and discourage wanting to smoke more if they focused on lung cancer (versus addiction) or contained images (versus text-only warnings). Finally, we also conducted exploratory analyses examining whether older adolescents, who are at greater risk of starting to smoke [16], responded differently to the warnings than younger adolescents.

## Materials and Methods

### Participants

During August and September 2010, boys ages 11–17 completed an online survey [17]. Their parents were members of a national panel of U.S. households constructed by Knowledge Networks through list-assisted, random-digit dialing supplemented by address-based sampling to capture cell phone-only households [18]. In exchange for participation in surveys, parents received points that they could later redeem for small cash payments. Households without Internet access received laptops and free Internet access. Boys received 5,000 points (worth about $5) for completing the survey. The survey company sent email invitations to participate in the study to 1,195 parents likely to have sons in the target age range. Among those who responded to their invitations (*n* = 752, 63%), 73% (*n* = 547) were eligible and completed the parent survey. We asked them to allow their adolescent sons to also participate. In households with more than one son age 11–17, we chose the son with the most recent birthday. Of the 547 parents, 421 (77%) had adolescent sons who also completed surveys. For the present analysis, we report data from 386 adolescent males, having excluded 35 adolescents who reported smoking at the time of the study (8% of the sample).

### Ethics statement

The Institutional Review Board at the University of North Carolina approved the study. Parents provided written consent, and sons provided written assent.

### Procedure

**Figure 1:**
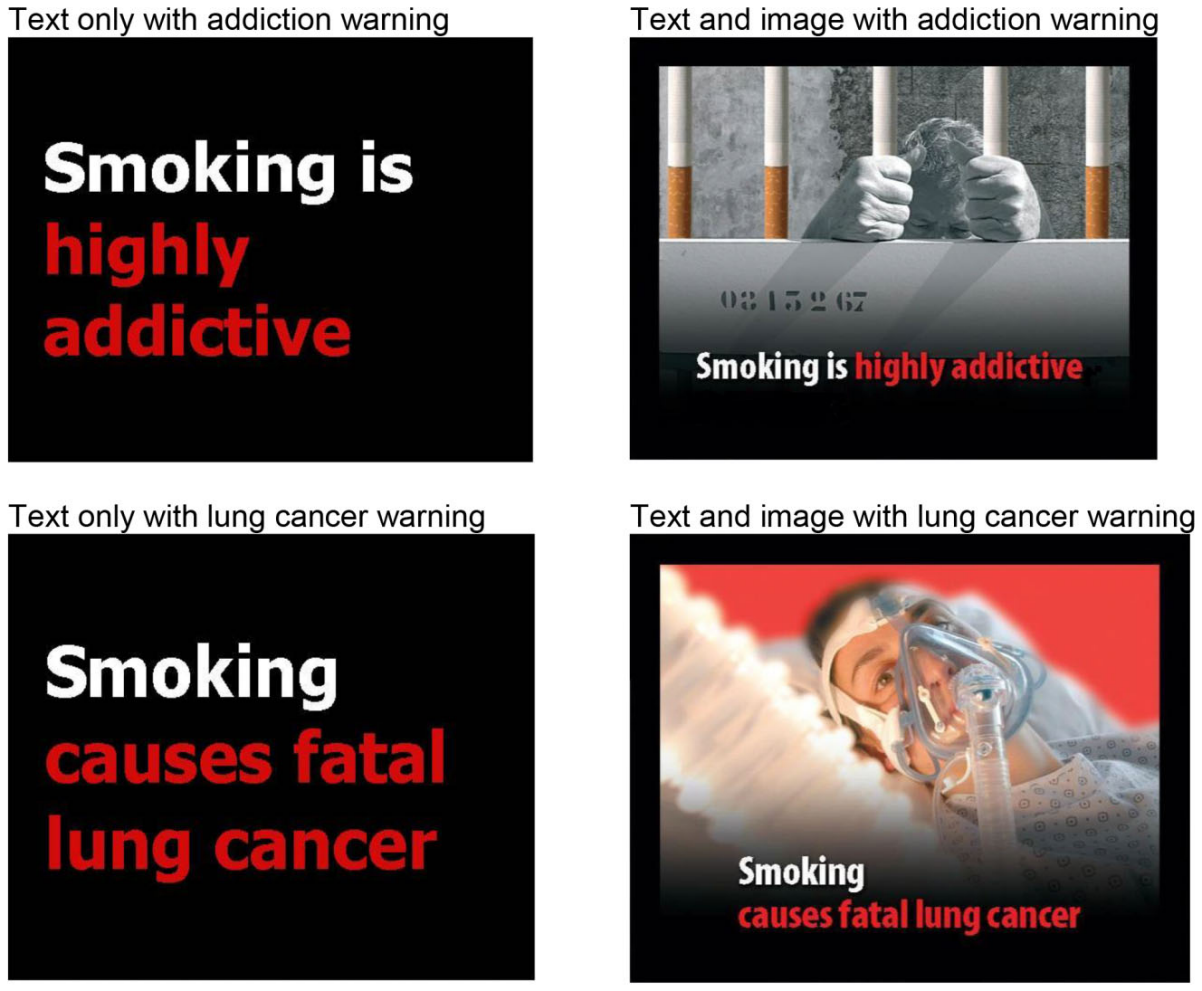
Cigarette warnings. Source: ec.europa.eu/health/archive/ph_determinants/life_style/tobacco/documents/uk_pictures.pdf.

Survey software randomly assigned sons to view a cigarette warning (Figure 1) in one of four conditions in a 2×2 between-subjects factorial experiment. We refer to these factors throughout the paper as “harm type” (addiction versus lung cancer) and “imagery” (text-only versus text with an image). We varied harm type by stating on the warning either “Smoking is highly addictive” (harm of addiction) or “Smoking causes fatal lung cancer” (harm of cancer). We chose these two harms because most adolescents know that smoking causes addiction and lung cancer [19], [20], and we wished to focus on risk perceptions, rather than knowledge of harms. We varied imagery by presenting one of these statements alone (text-only condition) or with an image (text with image condition). Because we wished to evoke thoughts about personal harm, we selected images of recognizably suffering individuals. Specifically, we chose an image of a man behind prison bars made of cigarettes to evoke addiction and a man in a hospital bed breathing through a ventilator to evoke lung cancer. The selected images are both currently in use on European cigarette packs [21]. Participants were randomized to see only 1 of the 4 warnings (text-only with addiction, image with addiction, text-only with lung cancer, or image with lung cancer). We pretested the warnings and survey with a sample of 23 adolescent males, and they reported no difficulty understanding and completing the items. A manipulation check with young adults found that warnings with images were more vivid than text-only warnings (*p*<.05). However, the vividness of warnings with lung cancer did not differ from those with addiction. All of the warnings received equivalent ratings of their ability to elicit self-efficacy to not smoke.

### Measures

While viewing the randomly assigned warning, participants responded to the question, “How much does this discourage you from wanting to smoke cigarettes?” The item had a 5-point response scale labeled from “not at all” (coded as 1) to “very much” (5). After the warning was no longer on screen, participants answered three questions in random order about the perceived likelihood and imaginability (i.e., the ease of picturing the harmful consequence) of addiction and the same three questions also in random order about lung cancer. The likelihood questions specified whether this was what an expert might say or their own belief: “If you started smoking more than once a week, what do you think an expert would say are the chances that you would eventually [become addicted/develop lung cancer]?” and “Setting aside what an expert might say, tell us what you think is true for you. If you started smoking more than once a week, what do you think the chances really are that you would eventually [become addicted/develop lung cancer]?” The perceived likelihood items had a 5-point response scale that ranged from “almost no chance” (1) to “almost certain” (5). The questions about imaginability of harms read: “How easy or hard is it to imagine [being addicted to cigarettes/having lung cancer]?” The response scale ranged from “very easy” (1) to “very hard” (4). They answered questions about the likelihood and imaginability of both addiction and lung cancer in all 4 experimental conditions, regardless of the warning viewed.

Parent surveys assessed sons' age, health insurance status, race (white or non-white), and ethnicity (Hispanic/Latino or non-Hispanic/Latino), as well as parents' marital status, education, and smoking habits. We classified parents as having “never smoked” (smoked less than 100 cigarettes in their lifetimes (*n* = 172) or refused to answer (*n* = 3)), being “former smokers” (smoked more than 100 cigarettes in their lifetimes but not current smokers), or being “current smokers” (smoke cigarettes some days or everyday). The survey also assessed household characteristics: income, urbanicity (as described by the Census Bureau definition of metropolitan statistical areas) [22], and region of residence (Northeast, Midwest, South, and West). Other than demographic questions answered by parents, our analyses are based on sons' responses. The complete parent and son surveys are available online [23].

### Data analyses

Preliminary analyses included linear regressions to identify demographic correlates of the seven dependent variables (discouragement from wanting to smoke and beliefs about the likelihood and imaginability of harms). Based on these analyses, we included race as a covariate in the remaining regression analyses because white race was bivariately associated with greater personal views of likelihood of addiction, greater expert views of likelihood of lung cancer, and lower imaginability of addiction (all *p's*<.05). Linear regression analyses modeled the main and two-way interaction effects of the two experimental factors (imagery and type of harm) and adolescent's age on the seven dependent variables. Analyses used age centered about the mean [24]. We present regression coefficients as standardized betas (*β*s).

We compared the imaginability of lung cancer and addiction using a paired *t*-test and then used within-subjects ANOVA to examine the effects of viewpoint (own perceived likelihood or perception of expert's view of likelihood) and harm (addiction or cancer) on likelihood estimates. We analyzed data with SPSS version 17.0 (SPSS Inc., Chicago, IL). Statistical tests were two-tailed with a critical alpha of 0.05.

## Results

Adolescents' mean age was 13.8 years (Table 1). Most were white (77%) and lived in urban areas (83%). About half of parents reported a household income of less than $60,000 (51%). Only 17% of parents were current smokers; most parents had never smoked (45%) or were former smokers (38%). Adolescent participants viewed the screen with the warning for a mean of 18 seconds (median 14 seconds). Our randomization check indicated only one of the ten sociodemographic characteristics (urbanicity) differed by condition (*p* = .04), but this characteristic was not associated with any of the dependent variables.

**Table 1:** Demographic Characteristics (*n* = 386).

	n (%)
**ADOLESCENT MALES**	
Age, mean (SD)	13.8 (2.1)
Health insurance	356 (92)
**Race**	
White	298 (77)
Non-white	88 (23)
**Ethnicity**	
Hispanic/Latino	65 (17)
Non-Hispanic/Latino	321 (83)
**PARENTS**	
**Marital status**	
Married/living with partner	317 (82)
Other	69 (18)
**Education**	
High school or less	175 (45)
Some college or more	211 (55)
**Smoking status**	
Never smoked	175 (45)
Former smoker	147 (38)
Current smoker	64 (17)
**HOUSEHOLDS**	
**Income**	
<$60,000	197 (51)
≥$60,000	189 (49)
**Urbanicity**	
Rural	67 (17)
Urban	319 (83)
**Region of residence**	
Northeast	70 (18)
Midwest	93 (24)
South	140 (36)
West	83 (22)

### Discouragement from wanting to smoke

**Figure 2:**
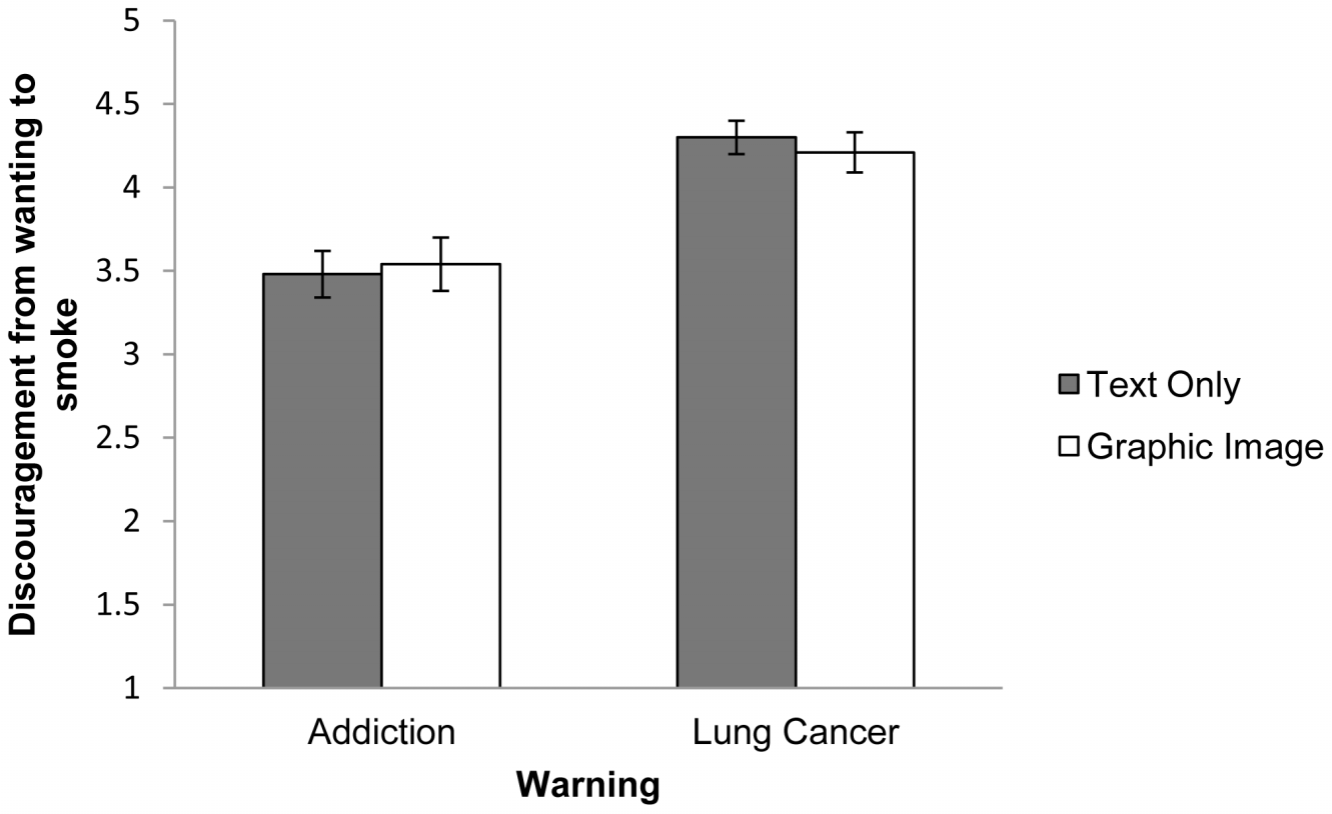
Effects of warnings on discouragement from wanting to smoke (*n* = 386). Error bars depict standard errors.

Most participants were “quite a bit” (20%) or “very much” (48%) discouraged from wanting to smoke by the warnings. As predicted, adolescents who viewed warnings with lung cancer messages were more discouraged than adolescents who viewed warnings with addiction messages (*β* = .28, *p*<.001) (Figure 2), with 60% being “very much” discouraged by lung cancer warnings versus 34% for addiction warnings. Contrary to prediction, there were no main or interaction effects of imagery (i.e., text with image versus text-only) on ratings of discouragement from wanting to smoke (Table 2). Age was not related to discouragement.

**Table 2:** Effects of warning imagery and respondent age (*n* = 386).

	Imagery^d^ (*β*)	Harm^e^ (*β*)	Imagery×Harm (*β*)	Age (*β*)	Age×Imagery (*β*)	Age×Harm (*β*)
Warning discourages you from wanting to smoke^a^	−.01	.28**	−.02	−.08	.04	.01
**Lung cancer**						
Perceived likelihood^b^: own	−.03	−.06	.03	−.07	.06	.03
Perceived likelihood^b^: expert	−.01	−.03	.04	−.07	.11*	.02
Imaginability^c^	−.08	.01	−.05	−.10*	−.01	.00
**Addiction**						
Perceived likelihood^b^: own	.02	−.01	−.02	−.07	.05	.07
Perceived likelihood^b^: expert	.04	.05	.01	−.07	.03	.11*
Imaginability^c^	−.01	−.01	.02	.03	−.02	−.06

* *p*<.05,

** *p*<.001.

*Note*: Analyses control for race. *βs* are standardized regression coefficients.

a Responses ranged from “not at all” (coded as 1) to “very much” (5).

b Responses ranged from “almost no chance” (1) to “almost certain” (5).

c Responses ranged from “very easy” (1) to “very hard” (4).

d Text-only (−1) versus image with text (1).

e Addiction (−1) versus lung cancer (1).

### Lung cancer

#### Perceived likelihood of lung cancer

The majority of participants rated their personal chance of developing lung cancer as “high chance” (38%) or “almost certain” (21%) if they were to start smoking at least once a week, and they believed an expert would rate their chance of developing lung cancer similarly (44% and 25%, respectively). Neither imagery (i.e., text with image versus text-only) nor harm type (i.e., addiction versus cancer) affected adolescents' own perceptions of the likelihood of lung cancer (Table 2).

There was an interaction between age and adolescents' beliefs about an expert's view of the likelihood of lung cancer (*p* = .04). When viewing text-only warnings, adolescents' beliefs about what an expert would say about their chance of getting cancer decreased with age (*p* = .02). In contrast, when viewing warnings with images, their beliefs about what an expert would say about their likelihood of developing cancer did not change with age (*p* = .56).

#### Imaginability of lung cancer

The majority of adolescent males found lung cancer to be “sort of hard” (30%) or “very hard” (46%) to imagine. Neither the type of harm nor the presence of imagery impacted the imaginability of lung cancer. Older adolescents found lung cancer to be more imaginable than did younger adolescents (*β* = −.10, *p* = .045).

### Addiction

#### Perceived likelihood of addiction

Most participants rated their chance of becoming addicted if they were to start smoking at least once per week as “high chance” (32%) or “almost certain” (35%) and believed that an expert would say they had “high chance” (35%) or an “almost certain” (44%) chance. There were no main or interaction effects of imagery or harm type on likelihood beliefs.

However, there was an interaction between harm type and age of the adolescents (*p* = .04). When viewing warnings with addiction messages, perceptions of an expert's estimates of addiction likelihood decreased with age (*p* = .02), whereas when viewing warnings with lung cancer messages, these perceptions did not change with age (*p* = .44). There was no interaction between imagery and age.

#### Imaginability of addiction

Most participants found addiction to be “sort of hard” (22%) or “very hard” (37%) to imagine. There were no effects of harm type, imagery, or age on the imaginability of addiction.

### Within-subjects analysis of perceived likelihood and imaginability of harms

Contrary to our prediction, participants found it harder to imagine having lung cancer than becoming addicted (Table 3) (means 3.09 vs. 2.72, *t* = −5.86, *p*<.001). Participants believed that they were more likely to become addicted than develop lung cancer (means 3.98 vs. 3.72, F (df 1, 385) = 29, *p*<.001). Participants also believed that experts would rate them as more likely to experience any harm than they would rate themselves (means 3.97 vs. 3.72, F (df 1, 385) = 59, *p*<.001). There was no interaction effect of role (own versus expert rating) and harm type on perceived likelihood beliefs.

**Table 3:** Mean (SD) discouragement from wanting to smoke, perceived likelihood, and imaginability (*n* = 386).

	Overall	Text+Addiction	Text+Cancer	Image+Addiction	Image+Cancer
Warning discourages you from wanting to smoke^a^	3.9 (1.3)	3.5 (1.4)	4.3 (1.1)	3.5 (1.4)	4.2 (1.2)
**Lung cancer**					
Perceived likelihood^b^: own	3.6 (1.1)	3.7 (1.1)	3.6 (1.1)	3.6 (1.0)	3.6 (1.1)
Perceived likelihood^b^: expert	3.8 (1.0)	3.9 (1.0)	3.8 (1.0)	3.8 (0.9)	3.8 (1.0)
Imaginability^c^	3.1 (1.0)	3.1 (1.0)	3.2 (0.9)	3.1 (1.1)	3.0 (1.0)
**Addiction**					
Perceived likelihood^b^: own	3.8 (1.2)	3.8 (1.2)	3.8 (1.3)	3.9 (1.2)	3.8 (1.1)
Perceived likelihood^b^: expert	4.1 (1.0)	4.0 (1.1)	4.1 (1.0)	4.1 (1.0)	4.2 (1.0)
Imaginability^c^	2.7 (1.2)	2.8 (1.2)	2.7 (1.2)	2.7 (1.3)	2.7 (1.2)

a Responses ranged from “not at all” (coded as 1) to “very much” (5).

b Responses ranged from “almost no chance” (1) to “almost certain” (5).

c Responses ranged from “very easy” (1) to “very hard” (4).

## Discussion

Common sense suggests that frightening images should scare kids away from smoking. However, in this sample, warnings with these graphic images did not discourage adolescent males from wanting to smoke more than text-only warnings. Images also did not increase adolescents' perceived risk of smoking's harms.

Past experiments and survey studies have typically found warnings with graphic images to have more impact than text-only warnings [8]. For example, in one within-subjects experiment, young adults were more discouraged from smoking by graphic warning labels with images than by text-only labels [25]. However, respondents in that study were older (ages 18–24) than participants in the present study and were current or former smokers, suggesting that they may have been less naïve about smoking and therefore had different responses to the warnings. Another study that used a design (a between-subjects experiment) and population (non-smoking adolescents) similar to our study found that text-only warning labels were more effective for reducing non-smoking American teenagers' intentions to smoke, while labels with images were more effective for non-smoking teenagers from Canada, which had already adopted such labels [9].

Potential reasons why warnings with images were not more effective in our study than warnings without them include that participants in our study were young (less than 17 years of age) and non-smokers. Because of their youth or lack of smoking experience, they may have had fixed, non-nuanced beliefs about the dangers of tobacco use that inclusion of imagery could not change. The null findings could also reflect the brief period of the exposure (i.e., the warning appeared on only one screen) or our choice of images. Not all pictorial health warnings are equally effective [26]–[28]. The images we chose were not gruesome, and gruesome images appear to be more effective in motivating young adult smokers to quit or not initiate smoking [29]. Further, neither image was an explicit image of diseased body part (e.g., mouth ulcers), and such images appear to be more effective than either symbolic images (like on the addiction warning) or images of human suffering (like on the lung cancer warning) [26], [28].

We also hypothesized that warnings focused on the harm of lung cancer would affect adolescents more than warnings focused on addiction. We found partial support for this hypothesis. Perceptions of the likelihood of lung cancer and addiction did not differ by condition. However, lung cancer-focused warnings discouraged participants from wanting to smoke more than addiction warnings. Cancer is widely feared. Holland and Cullen describe “cancerophobia,” a dysfunctional fear of the “five D's” of cancer: death, disfigurement, disability, dependence, and disruption of key relationships [30]. Cancer is also more concrete and perhaps easier to understand than addiction. Moreover, cancer is deadly, while addiction has to cause other sequelae in order to be deadly. Regardless of warning viewed, participants found it easier to imagine addiction than lung cancer overall, perhaps because they had previously seen people addicted to nicotine, but not people with lung cancer, in the media or among friends and family. That imaginability of lung cancer increased with age lends credence to the exposure explanation.

Respondents believed that their actual risk of harm was lower than what an expert would say, a finding that suggests unrealistic optimism [31]. If experts' opinions reflect average people's risk of harm, believing oneself to be at lower risk than what an expert would say is consistent with a self-enhancing optimistic bias (i.e., the participant believes he is luckier, healthier, or has more willpower than the average person) [32]. Alternatively, participants might have felt that experts were overstating their risk of harm to scare them away from smoking.

We suspect that beliefs about one's own risk may be more closely held and less changeable than beliefs about expert opinions of risk. One implication is that changes to expert risk beliefs may be easier to achieve. The impact of image-based warnings on experts' likelihood of developing cancer remained constant across ages, while for text-only warnings, experts' risk beliefs about lung cancer were lower among older respondents. Younger adolescents may view text-only messages as strict, authoritative statements that reflect expert opinions that smoking leads to high risk of lung cancer, but the tendency to view messages in this way decreases with age. Viewing “stronger” warnings (those with images), rather than “weaker” (text-only) ones, may counter this tendency for older adolescents.

Participants' perceptions of what an expert would say about their chance of becoming addicted also varied by age and warning type. Although their perceptions of what an expert would say about their chance of addiction decreased with age when viewing warnings about addiction, it remained constant for lung cancer warnings. One possibility is that adolescent males' views about experts' opinions and average likelihoods become more nuanced with age: they no longer view addiction as completely inevitable, or they can see how experts might be fallible. Respondents only engaged in this thinking about their chances of addiction when the warning they saw was on-target (i.e., related to addiction). Off-target warnings (i.e., related to lung cancer) did not affect these perceptions, perhaps because they did not induce deeper cognitive processing about the likelihood of addiction.

This study benefited from the use of a sample of adolescents from all regions of the country with proportions of white race and Hispanic/Latino ethnicity similar to the national average [33]. In addition, the experimental design permits causal inference, although conducting the experiment at a single time point did not enable us to assess whether discouragement led to future abstinence from smoking. However, non-smoking adolescents' decisions to avoid smoking strongly predict their later smoking behavior [34]. A limitation of the study is that participants saw cigarette warnings online as freestanding images rather than on cigarette pack silhouettes viewed online or on actual cigarette packs handled in person. Additionally, they only viewed the warnings while answering one item, so exposure was lower than what it would be in a naturalistic setting. While studies using actual cigarette packs are needed to confirm our findings, especially given that pack features can impact responses to warning labels [35], they may be hard to conduct with a national sample as we interviewed in the present study. Finally, future studies may derive some benefit from including more than two exemplar warnings per manipulation in order to confirm that the present findings about these particular lung cancer and addiction warnings generalize to other lung cancer and addiction warnings, including those ultimately selected by the FDA for future use.

The implementation of graphic cigarette warning labels is currently on hold. In November 2011, a U.S. District Judge blocked the FDA from requiring tobacco companies to put the previously selected labels on cigarette packages [5], and in April 2013, the FDA announced that they would not appeal this decision [6]. This announcement means that the FDA will need to select new graphic labels to meet the requirements of the Family Smoking Prevention and Tobacco Control Act [6], [7]. Understanding how adolescents at risk of smoking respond to different warnings can inform the future research needed to develop new warning labels. It is promising that, after viewing cigarette warnings, adolescent males were very discouraged from wanting to smoke and perceived high likelihood of experiencing harm. However, our study suggests the importance of having warnings that focus on cancer over addiction because cancer warnings were more discouraging and greater exposure to images of cancer could improve the imaginability of this serious potential harm. Ultimately, graphic warning labels on cigarette packs could be a valuable addition to other strategies shown to help prevent youth smoking, including mass media campaigns, cigarette taxes, clean air laws, age limits on purchasing, and advertising bans [15].

## References

[1] World Health Organization (2011) WHO Report on the Global Tobacco Epidemic, 2011: Warning about the Dangers of Tobacco. Geneva: World Health Org.

[2] World Health Organization (2003) WHO Framework Convention on Tobacco Control. Geneva: World Health Org.

[3] World Health Organization Tobacco Free Initiative (2009) Health warnings on tobacco products – worldwide, 2007. MMWR Morb Mortal Wkly Rep 58 19: 528–529. 19478720

[4] U.S. Food and Drug Administration (FDA) Cigarette health warnings. Available: http://www.fda.gov/TobaccoProducts/Labeling/Labeling/CigaretteWarningLabels/default.htm. Accessed 2012 5 September.

[5] R.J. Reynolds Tobacco Company, et al. v. United States Food and Drug Administration, et al., Civil Case 11-1482 (2011) United States District Court, District of Columbia.

[6] Koh HK (2013) A steadfast commitment to end the tobacco epidemic. Huffington Post. Available: http://www.huffingtonpost.com/dr-howard-k-koh/a-steadfast-commitment-to_b_2901521.html. Accessed 15 April 2013.

[7] Family Smoking Prevention and Tobacco Control Act of 2009. Pub. L. No. 111-31, Section 201, 123 Stat. 1842–1845. Available: http://www.fda.gov/TobaccoProducts/GuidanceComplianceRegulatoryInformation/ucm298595.htm. Accessed 2013 15 April.

[8] Hammond D (2011) Health warning messages on tobacco products: a review. Tob Control 20: 327–337. 21606180 10.1136/tc.2010.037630

[9] Sabbane LI, Lowrey TM, Chebat J (2009) The effectiveness of cigarette warning label threats on nonsmoking adolescents. J Consumer Aff 43 2: 332–345.

[10] Steinberg L, Graham S, O'Brien L, Woolard J, Cauffman E, et al (2009) Age differences in future orientation and delay discounting. Child Dev 80 1: 28–44. 19236391 10.1111/j.1467-8624.2008.01244.x

[11] DiFranza JR, Rigotti NA, McNeill AD, Ockene JK, Savageau JK, et al (2000) Initial symptoms of nicotine dependence in adolescents. Tob Control 9: 313–319. 10982576 10.1136/tc.9.3.313 PMC1748379

[12] Reyna VF, Farley F (2006) Risk and rationality in adolescent decision making. Psychol Sci Public Interest 7 1: 1–44. 26158695 10.1111/j.1529-1006.2006.00026.x

[13] Steinberg L (2005) Cognitive and affective development in adolescence. Trends Cogn Sci 9 2: 69–74. 15668099 10.1016/j.tics.2004.12.005

[14] Chassin L, Presson CC, Rose JS, Sherman SJ (2001) From adolescence to adulthood: age-related changes in beliefs about cigarette smoking in a Midwestern community sample. Health Psychol 20 5: 377–386. 11570652 10.1037//0278-6133.20.5.377

[15] U.S. Department of Health and Human Services (2012) Preventing Tobacco Use Among Youth and Young Adults: A Report of the Surgeon General. Atlanta, GA: U.S. Department of Health and Human Services, Centers for Disease Control and Prevention, National Center for Chronic Disease Prevention and Health Promotion, Office on Smoking and Health.

[16] Altman DG, Levine DW, Coeytaux R, Slade J, Jaffe R (1996) Tobacco promotion and susceptibility to tobacco use among adolescents aged 12 through 17 years in a nationally representative sample. Am J Public Health 86 11: 1590–1593. 8916525 10.2105/ajph.86.11.1590 PMC1380694

[17] Reiter PL, McRee AL, Kadis JA, Brewer NT (2011) HPV vaccine and adolescent males. Vaccine 29 34: 5595–5602. 21704104 10.1016/j.vaccine.2011.06.020 PMC3143221

[18] Dennis JM. Description of within-panel survey sampling methodology: the Knowledge Networks approach. Available: http://www.knowledgenetworks.com/ganp/reviewer-info.html. Accessed 2013 15 April.

[19] Arnett JJ (2000) Optimistic bias in adolescent and adult smokers and nonsmokers. Addict Behav 25 4: 625–632. 10972456 10.1016/s0306-4603(99)00072-6

[20] Weinstein ND, Slovic P, Waters E, Gibson G (2004) Public understanding of the illnesses caused by cigarette smoking. Nicotine Tob Res 6 2: 349–355. 15203808 10.1080/14622200410001676459

[21] United Kingdom cigarette warning labels. Available: ec.europa.eu/health/archive/ph_determinants/life_style/tobacco/documents/uk_pictures.pdf.

[22] U.S. Census Bureau. Glossary. Available: http://factfinder.census.gov/home/en/epss/glossary_r.html). Accessed 2013 15 April.

[23] Brewer NT. UNC Gillings School of Global Public Health, Health Cognition and Behavior Lab, HPV Research and Surveys. Available: http://www.unc.edu/~ntbrewer/hpv.htm. Accessed 2013 15 April.

[24] West SG, Aiken LS, Krull JL (1996) Experimental personality designs: analyzing categorical by continuous variable interactions. J Pers 64 1: 1–48. 8656311 10.1111/j.1467-6494.1996.tb00813.x

[25] O'Hegarty M, Pederson LL, Nelson DE, Mowery P, Gable JM, et al (2006) Reactions of young adult smokers to warning labels on cigarette packages. Am J Prev Med 30 6: 467–473. 16704939 10.1016/j.amepre.2006.01.018

[26] Cameron LD, Pepper JK, Brewer NT (2013) Responses of young adults to graphic warning labels for cigarette packages. Tob Control In press. 10.1136/tobaccocontrol-2012-050645 PMC3884029 23624558

[27] Glock S, Müller BCN, Ritter SM (2012) Warning labels formulated as questions positively influence smoking-related risk perception. J Health Psychol 18 2: 252–262. 22419415 10.1177/1359105312439734

[28] Hammond D, Thrasher J, Reid JL, Driezen P, Boudreau C, et al (2012) Perceived effectiveness of pictorial health warnings among Mexican youth and adults: a population-level intervention with potential to reduce tobacco-related inequities. Cancer Causes Control 23: 57–67. 22362058 10.1007/s10552-012-9902-4 PMC4586036

[29] Berg CJ, Thrasher JF, Westmaas JL, Buchanan T, Pinsker EA, et al (2011) College student reactions to health warning labels: sociodemographic and psychosocial factors related to perceived effectiveness of different approaches. Prev Med 53 6: 427–430. 21945706 10.1016/j.ypmed.2011.09.006 PMC3230734

[30] Holland R, Cullen A (1986) New insights and attitudes. In: Holleb AI, editors. The American Cancer Society cancer book. New York, NY: Doubleday.

[31] Weinstein ND (1987) Unrealistic optimism about susceptibility to health problems: conclusions from a community-wide sample. J Behav Med 10 5: 481–500. 3430590 10.1007/BF00846146

[32] Chambers JR, Windschitl PD (2004) Biases in social comparative judgments: the role of nonmotivated factors in above-average and comparative-optimism effects. Psychol Bull 130 5: 813–838. 15367082 10.1037/0033-2909.130.5.813

[33] U.S. Census Bureau. USA State & County Quickfacts. Available: http://quickfacts.census.gov/qfd/index.html. Accessed 2012 5 September.

[34] Pierce JP, Choi WS, Gilpin EA, Farkas AJ, Merritt RK (1996) Validation of susceptibility as a predictor of which adolescents take up smoking in the United States. Health Psychol 15 5: 355–361. 8891714 10.1037//0278-6133.15.5.355

[35] Wakefield M, Germain D, Durkin S, Hammond D, Goldberg M, et al (2012) Do larger pictorial health warnings diminish the need for plain packaging of cigarettes? Addiction 107 6: 1159–1167. 22372966 10.1111/j.1360-0443.2012.03774.x

